# Long noncoding RNAs are dynamically regulated during β-cell mass expansion in mouse pregnancy and control β-cell proliferation *in vitro*

**DOI:** 10.1371/journal.pone.0182371

**Published:** 2017-08-10

**Authors:** Giorgia Sisino, Alex-Xianghua Zhou, Niklas Dahr, Alan Sabirsh, Mangala M. Soundarapandian, Ranjan Perera, Erik Larsson-Lekholm, Maria Chiara Magnone, Magnus Althage, Björn Tyrberg

**Affiliations:** 1 Cardiovascular and Metabolic Diseases, Innovative Medicines and Early Development Biotech Unit, AstraZeneca, Mölndal, Sweden; 2 Institute of Biomedicine, University of Gothenburg, Gothenburg, Sweden; 3 Sanford Burnham Prebys Medical Discovery Institute, Orlando, Florida, United States of America; University of South Alabama Mitchell Cancer Institute, UNITED STATES

## Abstract

Pregnancy is associated with increased β-cell proliferation driven by prolactin. Long noncoding RNAs (lncRNA) are the most abundant RNA species in the mammalian genome, yet, their functional importance is mainly elusive. **Aims/hypothesis**: This study tests the hypothesis that lncRNAs regulate β-cell proliferation in response to prolactin in the context of β-cell mass compensation in pregnancy. **Methods**: The expression profile of lncRNAs in mouse islets at day 14.5 of pregnancy was explored by a bioinformatics approach, further confirmed by quantitative PCR at different days of pregnancy, and islet specificity was evaluated by comparing expression in islets versus other tissues. In order to establish the role of the candidate lncRNAs we studied cell proliferation in mouse islets and the MIN6 β-cell line by EdU incorporation and cell count. **Results**: We found that a group of lncRNAs is differentially regulated in mouse islets at 14.5 days of pregnancy. At different stages of pregnancy, these lncRNAs are dynamically expressed, and expression is prolactin dependent in mouse islets and MIN6 cells. One of those lncRNAs, Gm16308 (Lnc03), is dynamically regulated during pregnancy, prolactin-dependent and islet-enriched. Silencing Lnc03 in primary β-cells and MIN6 cells inhibits, whereas over-expression stimulates, proliferation even in the absence of prolactin, demonstrating that Lnc03 regulates β-cell growth. **Conclusions/interpretation**: During pregnancy mouse islet proliferation is correlated with dynamic changes of lncRNA expression. In particular, Lnc03 regulates mouse β-cell proliferation and may be a crucial component of β-cell proliferation in β-cell mass adaptation in both health and disease.

## Introduction

Compensatory mechanisms are present in adult β-cells to adapt to high metabolic physiological or pathophysiological demands, such as in pregnancy or obesity. One consequence is that β-cell mass increases through cell replication, neogenesis, and hypertrophy [1]. At homeostatic conditions β-cell mass is maintained via the balance between cell growth and apoptosis [2]. During pregnancy, insulin resistance increases [3] and β-cells respond by increasing mass to supply sufficient insulin. Failure of this adaptation process leads to gestational diabetes and to the concomitant risk of developing type 2 diabetes [4]. Studies in rodents have shown that the peak of β-cell proliferation during gestation coincides with increased circulating placental hormones [5], in particular prolactin. The molecular machinery that drives β-cell mass expansion in response to pregnancy and prolactin has been partly elucidated, which involves protein coding genes as well as microRNAs [6]. However, the role of long noncoding RNAs (lncRNAs) has never been explored.

LncRNAs are defined as non-protein coding transcripts longer than 200 nucleotides. They are located in intergenic regions or overlap with coding genes and are transcribed as antisense or sense RNAs [7]. LncRNAs typically are associated with gene regulation, acting on transcription, chromatin binding, or splicing [8], influencing biological processes such as the cell cycle [9], cell maturation [10], or organ development [11]. Loss of lncRNA regulation has been found to be the cause of several human diseases [12].

For the first time we here aim to identify lncRNAs that are dynamically regulated in islets during pregnancy and potentially are involved in β-cell mass compensation through regulation of proliferation. We identified islet lncRNAs that were differentially regulated during the peak of β-cell proliferation during pregnancy. We confirmed that six selected lncRNAs were dynamically expressed during pregnancy and partuition, and their regulation was driven by prolactin. In particular, we discovered a novel islet-enriched lncRNA Gm16308 (Lnc03) that functions as a positive regulator of β-cell proliferation. Although the Lnc03 human orthologue is so far not known it is likely that lncRNAs with equivalent functions exist in human β-cells. Hence, we believe that deepening the understanding of Lnc03 biology and its role in key β-cell signaling pathways could open the path for new β-cell regenerative therapies in diabetes.

## Materials and methods

### Animals

C57BL/6 female mice (12 weeks old; Charles River Laboratories) were maintained with 12-hour light/12 hour dark cycle; animals had *ad libitum* access to food and water. Control mice were defined as non-pregnant (NP) age-matched females. Starting day of pregnancy was defined when the vaginal plugs were verified. Pregnant mice were divided into 4 groups according to their days of pregnancy: 10.5 days (10.5D); 14.5 days (14.5D), 18.5 days (18.5D) and PP post partum (3–4 days after delivery). Islet isolation was performed in pregnant and control animals as described below. Brain, liver, thymus, kidney, adipose tissue and heart were harvested for RNA extraction from NP animals. All animal procedures followed the guidelines issued by the National Institutes of Health and protocols were approved by the local ethical committees.

### Ab initio assembly of the islet transcriptome from high coverage RNASeq data

Raw reads from high coverage mouse islet RNASeq were downloaded from GEO (GSE59285), trimmed with Trimmomatic to remove low-quality base reads and aligned against the mouse NCBI37/mm9 genome assembly with TopHat-2.0.4. A reference annotation was produced by combining mouse mRNAs in GenBank and gene predictions generated by Ensembl. The two annotations were downloaded from UCSC table browser and merged by removing all transcripts from the GenBank assembly that overlapped with any transcript in Ensembl and taking the union of the resulting annotations. This combined annotation was used as a reference to guide CuffLinks in ab initio construction of transcripts from the aligned reads. After removing transcripts on non-canonical chromosomes (M/Random/Un), the generated annotation contained 156,175 transcripts in 71,723 genes.

Raw reads of a low coverage RNASeq analysis of the islet transcriptome from NP and pregnant mice at gestational day 13–15 [13] were downloaded from GEO (GSE21860). The reads were aligned against the mouse NCBI37/mm9 genome assembly with TopHat. Aligned reads were then mapped to features in the ab initio assembly with HTSeq-count and finally DESeq was used to test for differential expression between groups.

### Microarray analysis of non-pregnant and pregnant mouse islets

Mouse islets were isolated from dams at gestational day 14.5, essentially as previously described [14]. Islets from 3–4 mice were pooled to collect at least 500 islets per sample. Three independent samples from NP and pregnant mice were collected. Immediately after isolation, total islet RNA was extracted with Stratagene RNA kit (Life Technologies). The cDNA library was built by SuperScript Plus Indirect RNA Amplification System with Oligo(dT)20 primers. Fragmented and labeled cDNA probes were hybridized with Invitrogen^™^ NCode^™^ non-coding RNA arrays. Microarrays were washed and scanned, and signal intensity was normalized for expression analysis (data can be downloaded from GEO, GSE100645). Primer sequences were BLASTed against a database generated from the transcript sequences of all transcripts in the ab initio annotation. Only hits that were unique and perfect (60 nt at 100% match) were considered valid. Two sample *t*-test was used to test for differential expression between groups. All in all, 22,958 genes in the ab initio assembly had at least one probe mapped and 706 transcripts were considered expressed above noise (baseline mean expression >5AU in at least one group) and differentially expressed at least 2-fold or differentially expressed with an adjusted P-value <0.05 (S1 Table).

### Mouse islet culture

Islets were hand-picked and processed immediately after isolation for total RNA extraction and qPCR analyses. To study expression of lncRNAs in response to recombinant mouse prolactin (Prl, R&D Systems), islets from control mice were pooled and left to recover overnight in RPMI 1640 (Gibco) supplemented with 11mM Glucose, 5% inactivated Horse serum (Gibco) and 1% of Penicillin/Streptomycin (Thermo Fisher Scientific). The day after, 30–35 islets were seeded per well in low adhesion plates, 200ng/ml or 500ng/ml Prl was added for 24 and 48 hours respectively, upon which islets were harvested for RNA extraction. To assess the efficiency of Lnc03 silencing and for proliferation assays, islets were isolated from eight 12 week old (5 female and 3 male) mice; islets were pooled and dispersed with Accutase (Gibco), 1ml per 1000 islets, and seeded in poly-l-lysine (Sigma Aldrich) coated plates [15]. Cells were seeded at a density of 25,000 cells in 96-well black plates for siRNA transfection followed by proliferation assays, and 40,000 cells in 48-well plates for siRNA transfection followed by RNA extraction.

### MIN6cl4 cell culture

The mouse β-cell line MIN6cl4 [16] between passages 24–35 were cultured in DMEM high glucose (Invitrogen) supplemented with 10% FBS, 1% P/S and 50μM β-mercaptoethanol (Sigma Aldrich). All experiments on MIN6 cells followed this procedure: the cells were starved 18h prior to all the treatments in DMEM low glucose (2.5mM glucose, Invitrogen), 50μM β-mercaptoethanol, 1% P/S and 0.1% BSA (Roche). Treatments with different compounds were performed in complete DMEM supplemented with 5% Horse Serum (Gibco). To study the effects of Prl on lncRNAs regulation, 200ng/ml or 500ng/ml Prl were used for 24h and 48h, respectively. To inhibit Stat5 in MIN6 cells 100μM Stat5 inhibitor (Nʹ-((4-Oxo-4H-chromen-3-yl)methylene) nicotinohydrazide, Cayman Chemicals) was added 1h prior to the 24h Prl treatment. Cells were finally harvested for RNA extraction and qPCR.

### RNA extraction and quantitative real-time PCR

Total RNA was isolated from islets and MIN6 cells using the RNAEasy Mini kit (Qiagen) according to the manufacturer’s protocol. Mouse organs were homogenized using Tissue Lyser II (Qiagen) before RNA extraction. DNA contaminants were eliminated using RNase-free DNase I (Qiagen). High Capacity cDNA kit (Invitrogen) was used to synthesize cDNA from 250ng to 1μg RNA, depending on the amount of available starting material. Quantitative real-time PCR was performed using Taqman Gene Expression Master mix (Applied Biosystems) according to the manufacturer’s instructions on QuantStudio^™^ 6 Flex Real-Time PCR (Applied Biosystems). Taqman probes (Thermo Fisher Scientific) were used for PCR quantification, as listed in S2 Table. The 2^-ddct^ method was applied to analyze gene expression [17]. Hprt (hypoxanthine phosphoribosyltransferase) was used as references gene. Data are expressed as fold change versus control (NP and non-treated samples).

### PCR array

Cell cycle gene expression profiling was assessed by using the Mouse Cell Cycle RT^2^ Profiler PCR Array (Qiagen). cDNA was prepared from 400ng total RNA per sample by using RT^2^ First Strand Kit (Qiagen). Quantitative real-time PCR was performed on the array using RT^2^ SYBR Green Mastermix (Qiagen) according to the manufacturer’s instructions on QuantStudio^™^ 6 Flex Real-Time PCR (Applied Biosystems). Data was normalized and analysed in GeneGlobe (Qiagen). The software measures and identifies the genes with the most stable expression via a non-normalized calculation. The C_T_ values for these genes are then geometrically averaged and used for the ΔΔC_T_ calculations (normalization genes were Gusb, Ppm1d, Tsg101, Hsp90ab1 and E2f3). Data are expressed as fold change versus control samples.

### SiRNA transfection and over expression

To study the effect of silencing Lnc03, we transfected MIN6 cells and dispersed mouse islets (Lipofectamine iRNAmax; Thermo Fisher Scientific) with AllStars Negative Control siRNA (Qiagen) or with custom designed siRNAs against Lnc03 (Ambion; sequences are reported in S3 Table), all according to the manufacturers’ protocols. Lnc03 overexpression assay was carryed out using plasmids pcDNA3.1 EmGfp (control) and pcDNA3.1 Lnc03. MIN6 cells were seeded in a 96-well plate at a density of 40,000 and transfected with 100ng of either plasmid using Lipofectamine Ltx with Plus reagent (Thermo Fisher) according to the manufacturer’s protocol. Cells were starved as described above and Prl at 500ng/ml was added for 5 days. For Edu incorporation and proliferation evaluation we used the same protocol as described above.

### Proliferation assays

Proliferation of MIN6 cells was assessed by cell count and EdU incorporation. MIN6 cells were transfected with negative control siRNA and siRNA against Lnc03, starved for 18h with DMEM 2.5mM glucose and 0.1%BSA, and finally incubated with 500ng/ml Prl for 72h. For cell count, cells were plated in 24-well plates at a density of 100,000 per well. After trypsinization, cells were counted with a Cedex HiRes Analyzer (Roche). For EdU incorporation, MIN6 cells were seeded in 96-well plates at a density of 25,000 cells per well and incubated for 2h with 10μM EdU. The EdU click-it kit (Thermo Fisher Scientific) was used according to the manufacturer’s protocol. The fraction of EdU positive cells was determined using an ImageXpress^®^ Micro High-Content Imaging System (Molecular Devices).

For mouse islet proliferation analyses, islets were dispersed and seeded in 96-well plates and transfected with siRNA against Lnc03 with and without Prl. Cells were starved for 18h with RPMI containing 5.6 mM glucose and 2.5% horse serum, followed by incubation in the same medium with the addition of 500ng/ml Prl for 72h. EdU was added during the last 24h of Prl treatment. Cells were fixed, and the EdU click it kit instructions were followed. The cells were stained with a guinea pig anti-insulin Ab (PA1-26938, Thermo Fisher) at 1:1000. Anti-guinea pig AlexaFluor549 was used as secondary antibody. Only cells positive for both EdU and insulin were considered for the analyses.

### Statistical analysis

Data are presented as mean±SEM. Statistical analyses were performed using the two-tailed paired Student's t-test or One-way ANOVA followed by Bonferroni post hoc test or Fisher’s exact test as appropriate (Graph Pad Prism 6.0, GraphPad Software).

## Results

### Identification of differentially expressed lncRNAs in mouse islets during pregnancy

**Fig 1:**
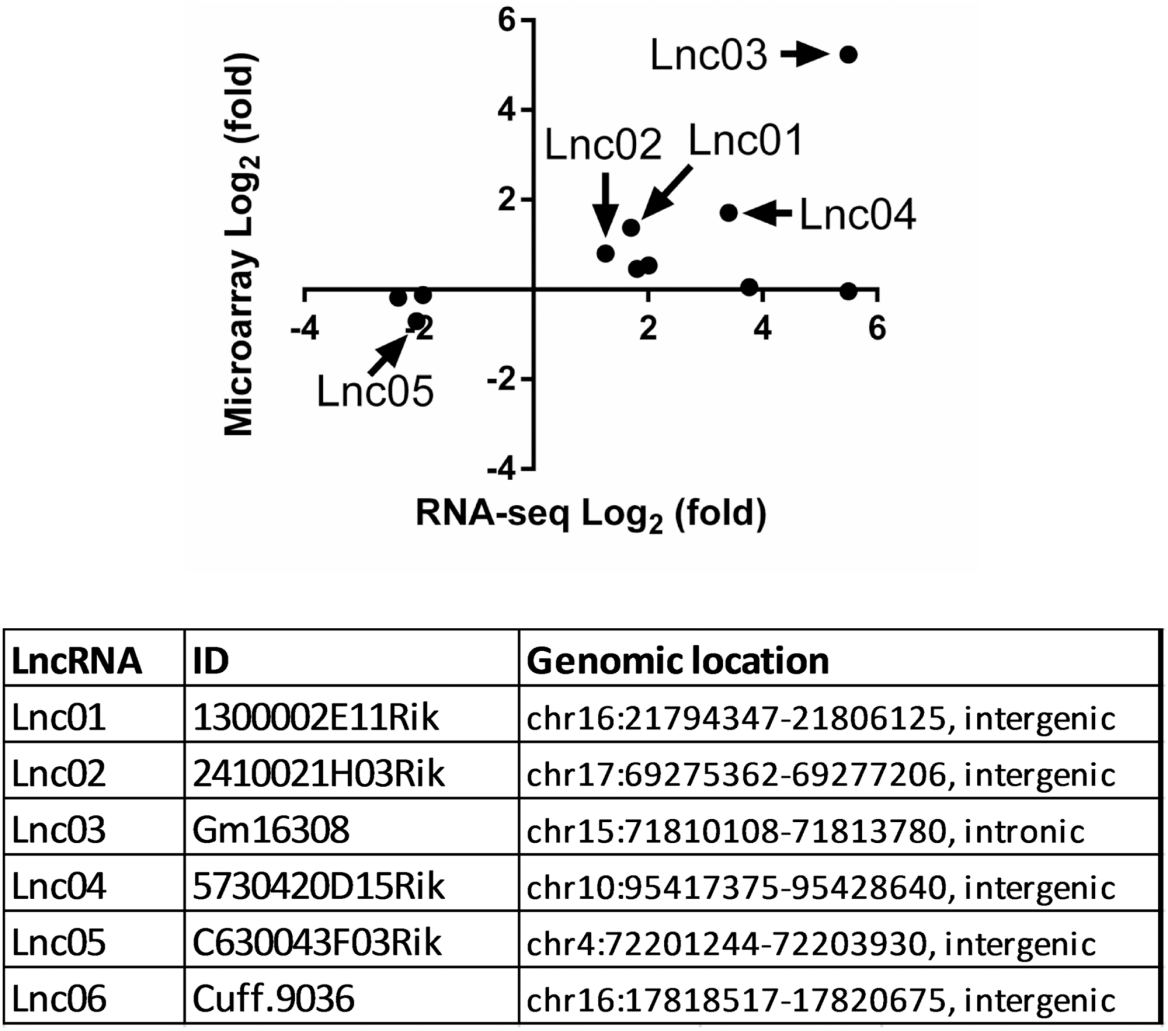
Identification of differentially expressed lncRNAs in mouse islets during pregnancy. Differentially expressed non-coding RNAs at gestational day 14.5 identified by RNA-seq analysis were filtered to remove rRNAs, snoRNAs, and miRNAs. The log2-transformed fold change of remaining ncRNAs is compared with microarray analysis. Five potential lncRNAs were selected. An additional lncRNA Cuff.9036 identified by Cufflinks (GeneBank accession MF497421), as a novel transcript only present in our annotation, was also selected for further study (table in Fig 1).

To identify differentially expressed lncRNAs in mouse islets during pregnancy at the reported peak of β-cell proliferation (5), we collected islets from non-pregnant (NP) female mice and pregnant mice at gestational day 14.5. The mouse islet transcriptome was analyzed by the NCode lncRNA microarray. Moreover, we reanalyzed published RNASeq data of the islet transcriptome in NP and pregnant mice at gestational day 14.5 [13]. Both microarray and RNA-seq data showed that Tph1 was the most significantly differentially expressed gene [13] (S1 Fig). However, according to our stringent statistical analysis, RNA-seq displayed higher sensitivity in detecting differentially expressed lncRNAs compared to the NCode array. Thus, we selected the lncRNAs with significantly differentiated expression as identified in RNA-seq data and examined their expression with the NCode array data (Fig 1, upper panel). A portion of these transcripts revealed concordant robust regulatory patterns between the two datasets in pregnant islets. Among these transcripts, additional criteria were applied for selection of the top five candidate lncRNAs (Lnc01-05, Fig 1: lower panel), including high relative expression and confidence in no protein-coding capacity. In addition, we identified one lncRNA (Lnc06, Fig 1: lower panel) in mouse islets that was not annotated before and showed a significant upregulation in pregnant islet (fold change 2.39, adjusted P-value = 8.57E-16, S1 Table).

### Regulation of lncRNAs expression during pregnancy

**Fig 2:**
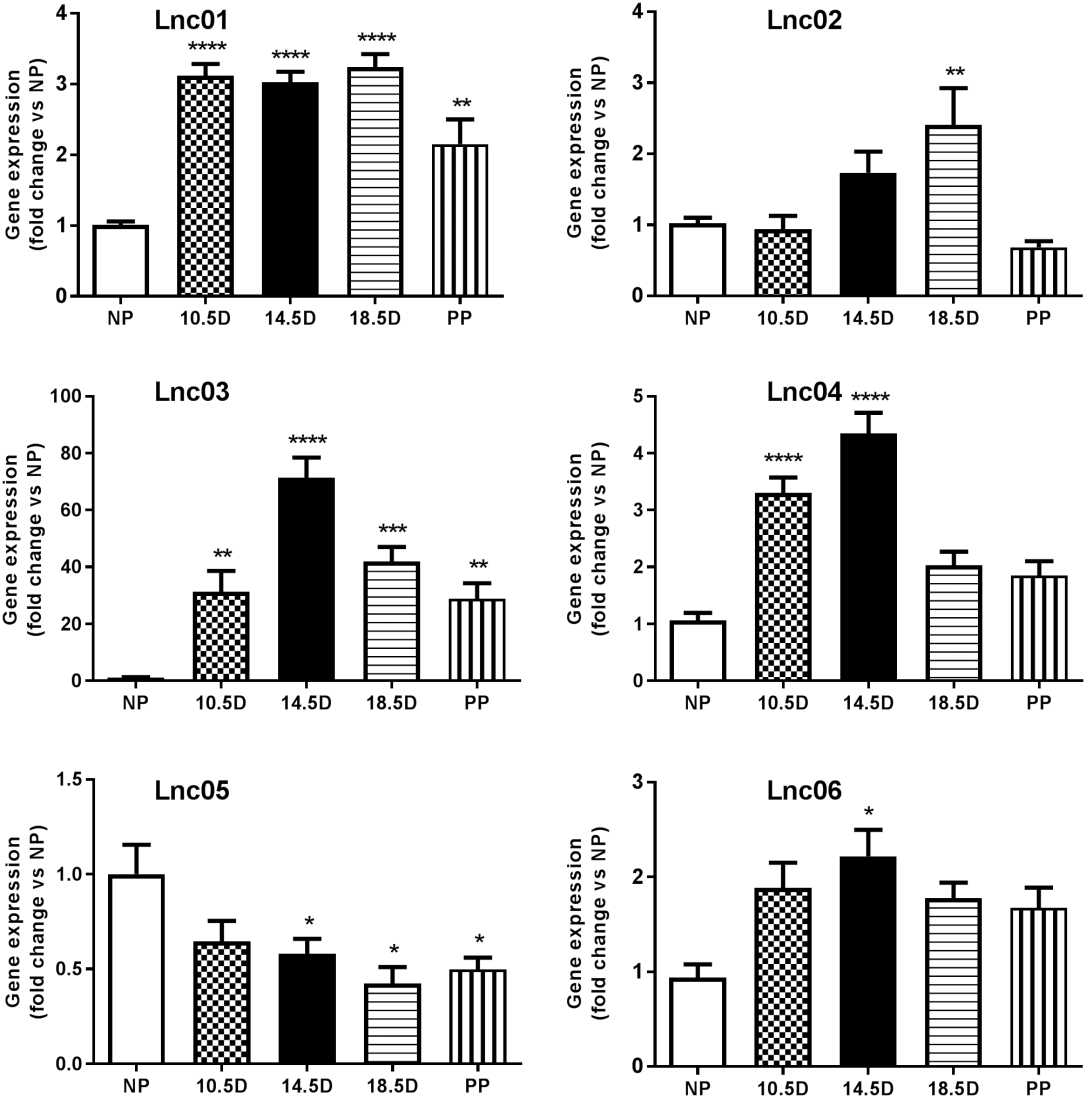
Expression of candidate lncRNAs in mouse islets at different stages of pregnancy. Total RNA was used to perform RT-qPCR of Lnc01-Lnc06. Data are presented as fold change versus non pregnant and mean±SEM, n = 4–8. NP (non-pregnant), 10.5D, 14.5D, 18.5D (days of pregnancy), and PP (post partum). Statistically significant differences were determined using One-way ANOVA with Bonferroni post hoc test. *p< 0.05; **p < 0.01; ***p < 0.001.

**Fig 3:**
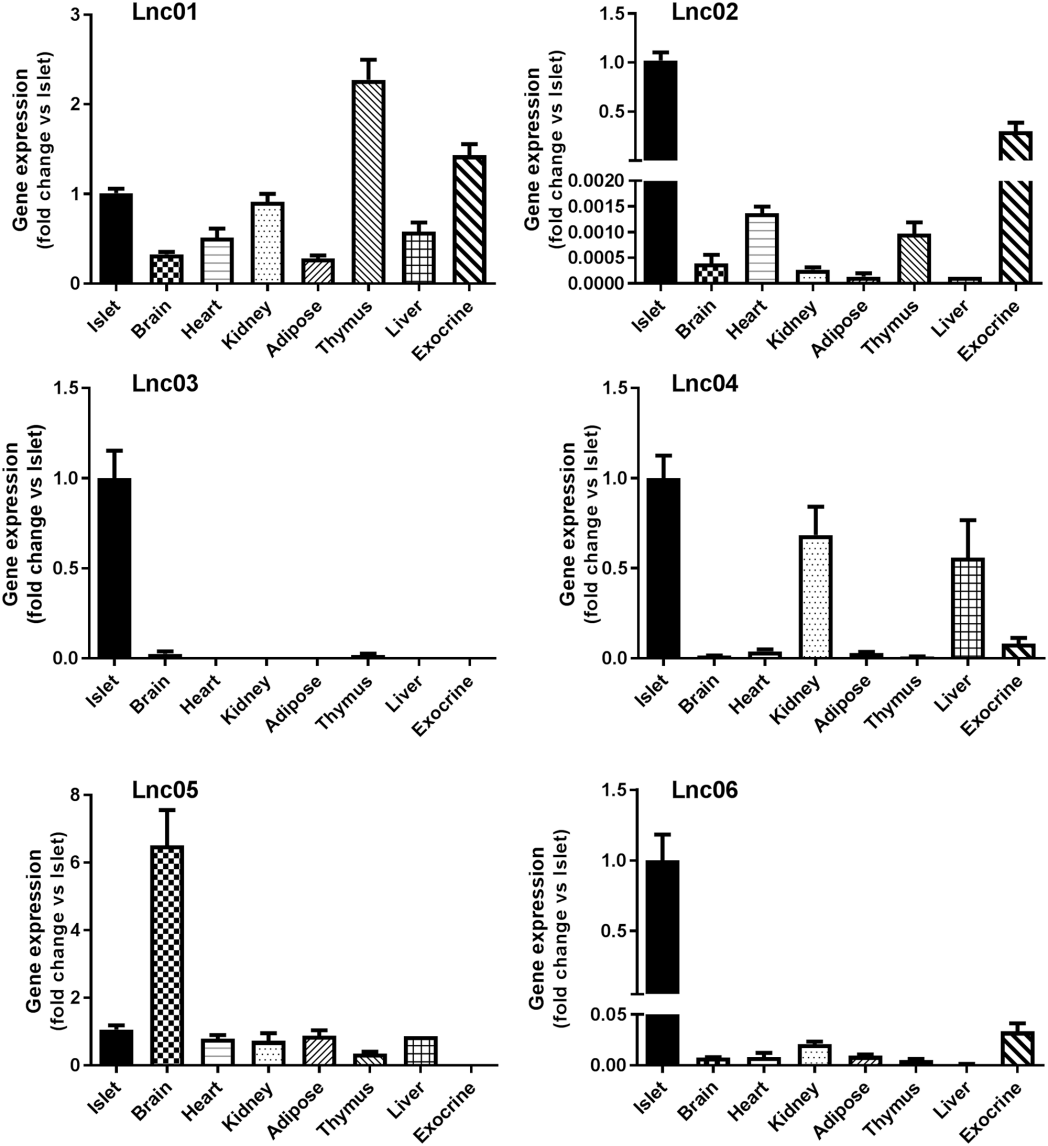
Expression of candidate lncRNAs in 8 different mouse tissues. Total RNA from control animals was used to perform RT-qPCR of Lnc01-Lnc06. The results were normalized to Hprt (hypoxanthine guanine phosphoribosyl transferase), and Gapdh (glyceraldehyde 3-phosphate dehydrogenase) expressed as fold change versus islets. Data are shown as mean±SEM, n = 4–8.

The expression pattern of candidate lncRNAs was confirmed by qPCR in islets isolated from mice at different stages of pregnancy. We found that Lnc01 expression was significantly increased compared to NP mice during all stages of pregnancy and after parturition (Fig 2). Lnc02 expression peaked slightly at 18.5D of gestation. The Lnc03 transcript was dramatically up-regulated throughout pregnancy and decreased postpartum, resembling a pattern comparable to the dynamics of β-cell mass adaptation. The expression of Lnc04 increased significantly at 10.5 and 14.5 days of gestation. Lnc05 appeared to be down-regulated slightly starting at 14.5 days of pregnancy, confirming our profiling studies (*cf*. Fig 1). Lnc06 was increased at all stages of pregnancy, but reaching statistical significance only at day 14.5. To investigate the islet specificity we analyzed expression of the lncRNAs in a selection of organs harvested from 12 weeks old NP mice. We analyzed brain, heart, kidney, adipose, thymus, and liver tissues, as well as isolated islets and pancreatic exocrine cells by qPCR (Fig 3). Lnc01, Lnc02, Lnc04 Lnc05 and Lnc06 did not appear to be islet-enriched, but they were also expressed in exocrine cells or in other tissues, although some were enriched in islets within the pancreas. On the other hand, Lnc03 was expressed almost exclusively in islets and totally absent in exocrine tissue. Comparing to public data in Expression Atlas (www.ebi.ac.uk), Lnc03 is also expressed in skeletal muscle and testis at about 5 times the expression levels of brain. This still suggests significant enrichment in islets (*cf*. brain in Fig 3).

### Upregulation of Lnc03 by Stat5-mediated Prl signaling

**Fig 4:**
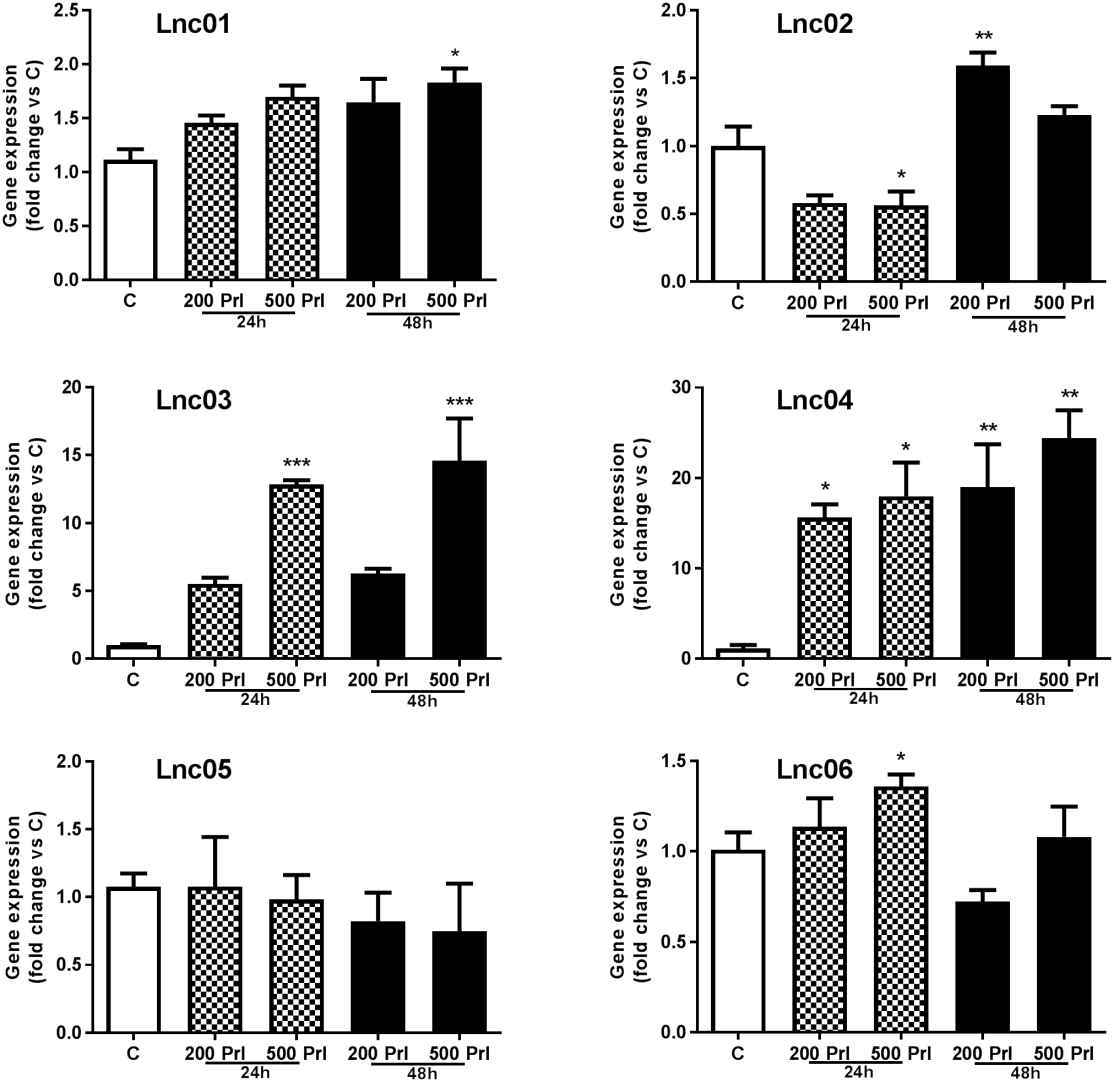
Expression of candidate lncRNAs in isolated mouse islets treated with Prl. Isolated mouse islets were treated with 200ng/ml or 500ng/ml (200 Prl, 500 Prl) for 24h or 48h; non-treated islets were used as control (C). Total RNA was used to perform RT-qPCR. Data are presented as fold change versus C, mean±SEM, n = 3. Statistically significant differences were determined using One-way ANOVA with Bonferroni post hoc test. *p< 0.05; **p < 0.01; ***p < 0.001.

**Fig 5:**
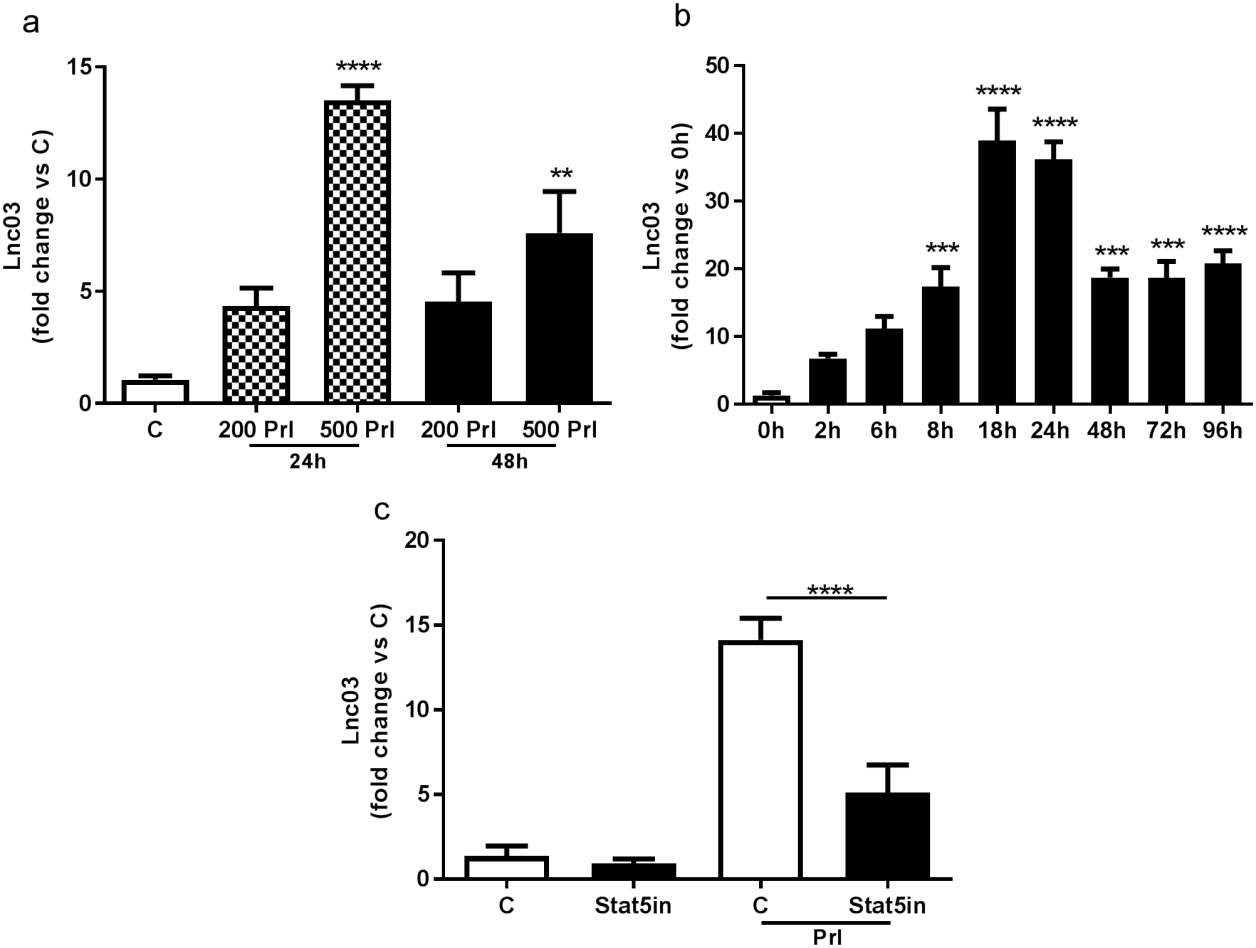
Analysis of Lnc03 expression in MIN6 cells. a) MIN6 cells were treated with 200 or 500ng/ml Prl (200 Prl, 500 Prl) for 24h (grey bars) and 48h (black bars), non-treated cells were used as control (C, white bar). Total RNA was used to perform RT-qPCR. Data are presented as fold change versus C, mean±SEM, n = 3. b) Lnc03 expression was evaluated in a time course experiments in MIN6 cell treated with 500ng/ml Prl (black bars). 0h (white bar) represents non-treated control cells. Data are presented as fold change versus 0h, mean±SEM, n = 4. c) MIN6 cells were treated with 100μm Stat5 inhibitor alone (Sta5in) or together with 500ng/ml Prl (Sta5in+Prl) or Prl alone for 24h. Control cells were not treated (C white bar). Data are presented as fold change versus C, mean±SEM, n = 4–8.

To evaluate the direct effect of Prl on the regulation of candidate lncRNA expression, we established an *in vitro* model using mouse islets isolated from non-pregnant mice treated with Prl. While most of the lncRNAs were weakly regulated depending on the time of treatment exposure and the concentration of Prl, only Lnc03 and Lnc04 were robustly positively regulated by Prl (Fig 4). From the results above, we concluded that Lnc03 was the only lncRNA that demonstrated dynamic regulation during pregnancy, islet enriched, as well as a robust regulation by Prl. Therefore, we focused further studies on Lnc03. We first confirmed that Lnc03 does not have any protein coding potential using NCBI OrfFinder [18] (not shown). For efficient in vitro exploration, we ascertained that regulation of Lnc03 expression in the mouse insulinoma cell line MIN6 matched the data observed in primary islets, and indeed, the expression of Lnc03 was dependent on Prl (Fig 5a and 5b). On the other hand, Lnc03 was not positively regulated by other proliferative stimuli (S2 Fig), suggesting that Prl signaling specifically activates Lnc03. We then explored if Lnc03 regulates Col22a1 since its location is on the opposite strand in an intronic region of that gene. However, Col22a1 expression was not regulated by Lnc03 knock down, nor by Prl (S3 Fig). We finally investigated if the expression of Lnc03 in response to Prl was dependent on Stat5 (Fig 5c), known to be a mediator of Prl stimulated β-cell proliferation during pregnancy [13,19,20]. Lnc03 was indeed regulated by Stat5.

### Lnc03 regulates β-cell proliferation

**Fig 6:**
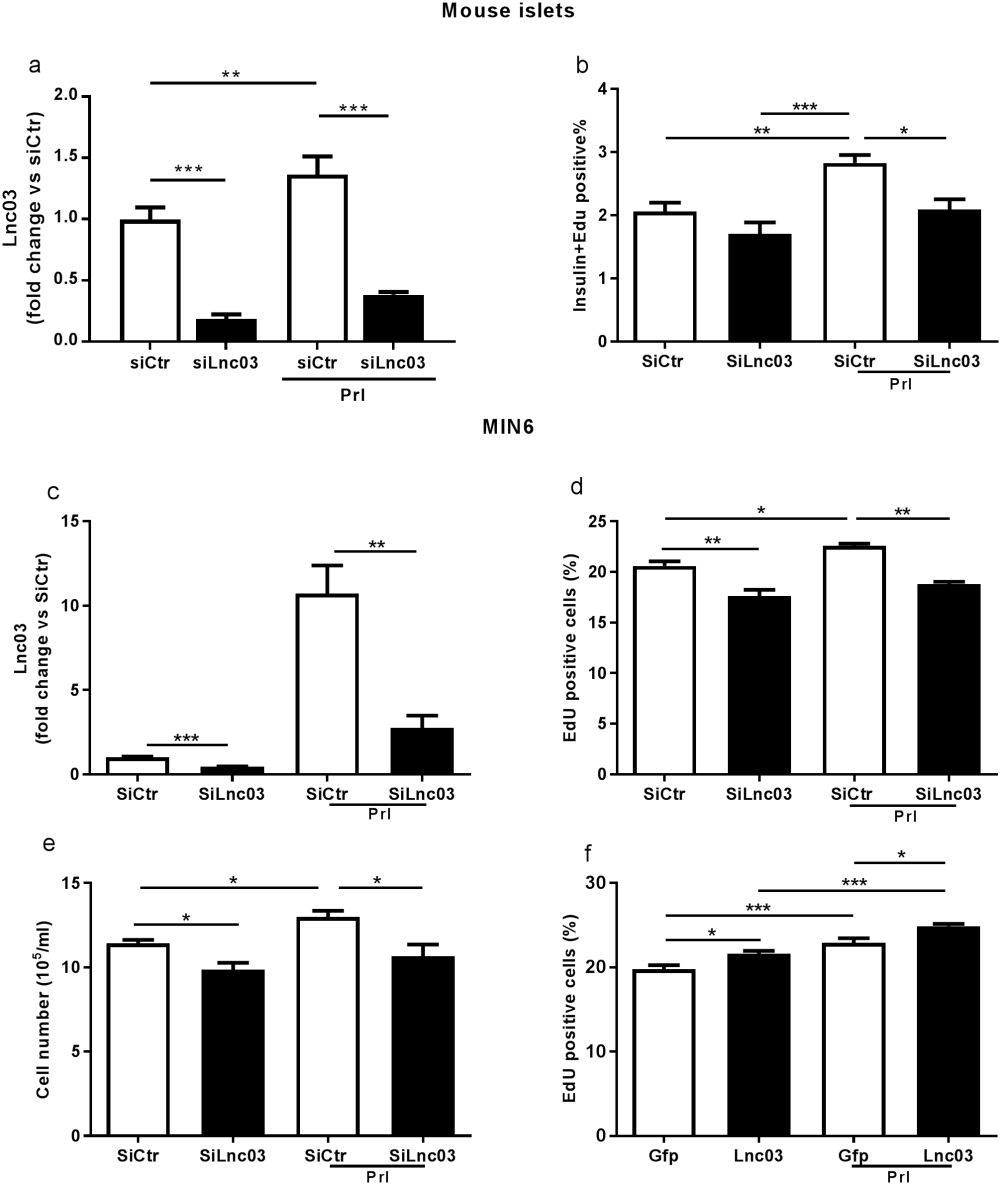
Lnc03 impacts proliferation in mouse β-cells and in MIN6 cells. a) Evaluation of Lnc03 expression by RT-qPCR in mouse islets dispersed after transfection with siRNA negative control (SiCtr, white bars) and with SiRNA against Lnc03 (SiLnc03, black bars), in the absence or presence of 500ng/ml Prl for 72h. Data are presented as mean±SEM, n = 3. b) Percentage of dispersed mouse islets positive for both Insulin and Edu after transfection with siRNA negative control (SiCtr, white bars) and with siRNA against Lnc03 (SiLnc03, black bars), in the absence or presence of 500ng/ml of Prl for 72h. Data are presented as mean±SEM, n = 6. c) MIN6 cells were transfected in the presence or absence of 500ng/ml Prl. Total RNA was extracted, and expression of Lnc03 was determined by RT-qPCR; data are presented as mean±SEM, n = 4. d) Percentage of MIN6 cells positive for EdU after transfection with SiRNA negative control and with SiRNA against Lnc03, with and without Prl. e) MIN6 cell count after transfection with and without Prl. Data are presented as mean±SEM, n = 4. f) Percentage of MIN6 cells positive for Edu after transfection with GFP (control) or Lnc03 plasmid, with and without Prl, data are presented as mean±SEM n = 6. Statistically significant differences were determined with One-way ANOVA with Bonferroni post hoc test. *p< 0.05; **p < 0.01; ***p < 0.001.

To assess the function of Lnc03 we transfected dispersed primary mouse islet cells and MIN6 cells with siRNA against Lnc03. We tested the efficiency of Lnc03 silencing using three different siRNAs. The most robust knockdown (~75%) was achieved with SiLnc03#1 (S4 Fig, Fig 6a). We then investigated if Lnc03 silencing directly impacts β-cell proliferation in MIN6cl4 cells and dispersed mouse islets with or without Prl. We observed that silencing Lnc03 suppressed β-cell proliferation (Fig 6b–6d). We then investigated if Lnc03 independently regulates proliferation or if it is dependent on Prl signaling. Interestingly, we observed that Lnc03 overexpression resulted in more cells incorporating EdU in both the presence and absence of Prl, suggesting that Lnc03 expression is sufficient to drive β-cell proliferation even without prolactin stimulation (Fig 6f).

To further confirm the role of Lnc03 in proliferation and to investigate if any particular group of genes were regulated by Lnc03 we knocked down Lnc03 in prolactin treated MIN6 cells and studied expression of cell cycle related genes with a qPCR array (S5 Fig). We could indeed confirm regulation of proliferation by downregulation of Mki67, a common marker for proliferating cells. In addition, we found that 19 out of 84 genes represented on the array were downregulated upon Lnc03 knock down, suggesting that the role of Lnc03 is to positively regulate cell cycle related genes, directly or indirectly. However, we did not find an overrepresentation of any particular group of genes that could have suggested a more specific mechanistic role for Lnc03 (Fisher’s exact test, not significant).

In conclusion, our findings suggest that Lnc03 expression in response to Prl is regulated downstream of Stat5, with an impact on β-cell proliferation, as an essential component of the Prl-dependent β-cell proliferation response. We also conclude that Lnc03 overexpression is sufficient to drive β-cell proliferation even in the absence of Prl.

## Discussion

Despite their lack of coding protein potential [21] lncRNAs are now considered key drivers of multiple biological processes [22]. The recent discovery of thousands of lncRNAs being islet or β-cell specific [23] points to the possibility that they are involved in novel mechanisms of islet biology, *e*.*g*. regulating insulin secretion or proliferation. In this study, we demonstrate that β-cell proliferation associated with pregnancy correlates with changes in the expression of a set of lncRNAs. Using microarray and RNASeq data we identify six lncRNAs that are up or down-regulated in mouse islets at 14.5 days of pregnancy, which corresponds to the peak of cell proliferation [24] and strongly correlates with the associated increase of circulating Prl [25]. During pregnancy circulating prolactin rises in parallel with increased β-cell mass, and its stimulatory effects on β-cell proliferation in vitro are well known [26]. We further analyze the dynamics of these lncRNAs in mouse islets before, during, and after gestation. We observe that Lnc03 expression is low in non-pregnant islets while its transcription peaks at gestational day 14.5 and then gradually decreases in the later phase of pregnancy and post partum. Consequently, the expression pattern of Lnc03 parallel β-cell mass adaptation during pregnancy [27], suggesting that it may be a regulator of this process. Interestingly, Lnc03 turns out to be islets-enriched when screening expression in seven other major tissues, which may mean that Lnc03 is a relatively unique regulator of endocrine cell proliferation. We utilize both primary mouse islets and MIN6 cells to elucidate the role of Lnc03 further in islet function. Although primary islets is a preferred model, we draw conclusions also from MIN6 cells based on their ease of manipulation and verify select key findings in primary islets. For example, plasmid transfection (over-expression of Lnc03) in primary β-cells to study proliferation was not feasible in our hands due to low efficiency of transfection. Among the six lncRNAs, we observe that Lnc03, in particular, is regulated by Prl, in vitro and ex vivo. Regulation of Lnc03 in response to prolactin was more pronounced in intact islets (5-15-fold, Fig 4) than in dispersed islet cells (2-fold, Fig 6a). This discrepancy is likely explained by the dispersion of the islets, which results in less responsive cells to many stimuli. Nevertheless, manipulating Lnc03 expression in both MIN6 and primary β-cells resulted in altered proliferation rates as expected if Lnc03 is part of the Prl-driven proliferation response. Downregulation with siRNA inhibited proliferation, whereas overexpression stimulated proliferation.

We conclude that Lnc03 regulation rely on Stat5-dependent components of the Prl activation pathway. Prl receptor activation in rodent β-cells triggers the Stat5 pathway [28,29]. We observe a strong decrease in Lnc03 expression when a Stat5 inhibitor was used together with Prl, suggesting that Lnc03 is under transcriptional regulation of activation pathways dependent on Stat5 signaling [30,31]. In MIN6 cells we observe that Lnc03 regulates proliferation also in the absence of Prl, whereas this was not apparent in primary β-cells. This is not surprising since components of Prl-signaling is not unique to Prl, and other basal proliferative stimuli in MIN6 cells may activate e.g. Stat5. Consequently, we cannot exclude that Lnc03 is also part of the basal proliferative machinery in β-cells, at least in MIN6, and that other proliferation stimuli yet to be identified would depend on Lnc03.

It is attractive from a therapeutic perspective to stimulate β-cell proliferation in diabetes. However, to be able to do that safely, either the target must be unique to β-cells or complicated strategies for tissue targeting must be employed to avoid drug exposure and proliferation in cells outside the islet. Up until now, very few, if any, such β-cell specific proliferating targets have been identified. Interestingly, the pattern of Lnc03 expression to some degree fits this profile with an islet enriched expression profile. Taken together our data point to Lnc03 being a regulator of proliferation at least in pregnancy. However, Lnc03 over-expression stimulates β-cell proliferation also in the absence of Prl, suggesting that a therapeutic upregulation approach to increase insufficient β-cell mass in diabetes would be possible.

LncRNA sequences often are not conserved phylogenetically [32]. Despite thorough efforts, we were not able to identify the Lnc03 human orthologue (not shown). However, some lncRNAs have functional orthologues [33] that could be unveiled by secondary structure analysis. Further investigations will be needed to identify which lncRNA corresponds to Lnc03 in humans. Understanding the molecular interaction partner(s) of Lnc03 would be of particular importance to clarify this, allowing exploration of the corresponding signalling complex in human cells.

## Conclusion

In summary, our findings demonstrate that Lnc03 plays a novel role in the regulation of β-cell proliferation. Although mouse specific at this stage, the identification of a novel lncRNA involved in regulation of β-cell proliferation, with a relative degree of islet specificity, is an important step towards understanding the full complexity of regulation of β-cell proliferation and mass adaptation in health and disease. It also gives evidence that cell-type enriched regulators are important in β-cell proliferation and, therefore, may be suitable drug targets that do not impact other cell types when identified in human.

## Supporting information

S1 Fig (PDF)

S2 Fig Lnc03 in response to growth factors. (PDF)

S3 Fig Col22a1 expression in MIN6. (PDF)

S4 Fig Validation of Lnc03 knockdown in MIN6 cells. (PDF)

S5 Fig Analyses of cell cell cycle genes after Lnc03 knock down. (PDF)

S1 Table NcRNA transcripts differentially expressed in microarray. (XLSX)

S2 Table Taqman probes. (PDF)

S3 Table SiRNA against Lnc03 sense sequences. (PDF)
