# Supplementary material for: Long noncoding RNAs are dynamically regulated during β-cell mass expansion in mouse pregnancy and control β-cell proliferation in vitro
Source: PLoS One. 2017 Aug 10;12(8):e0182371. doi: 10.1371/journal.pone.0182371 (PMC5552087; doi:10.1371/journal.pone.0182371)
Supplement: S1 Fig — (PDF) [file pone.0182371.s001.pdf]

S1 Fig

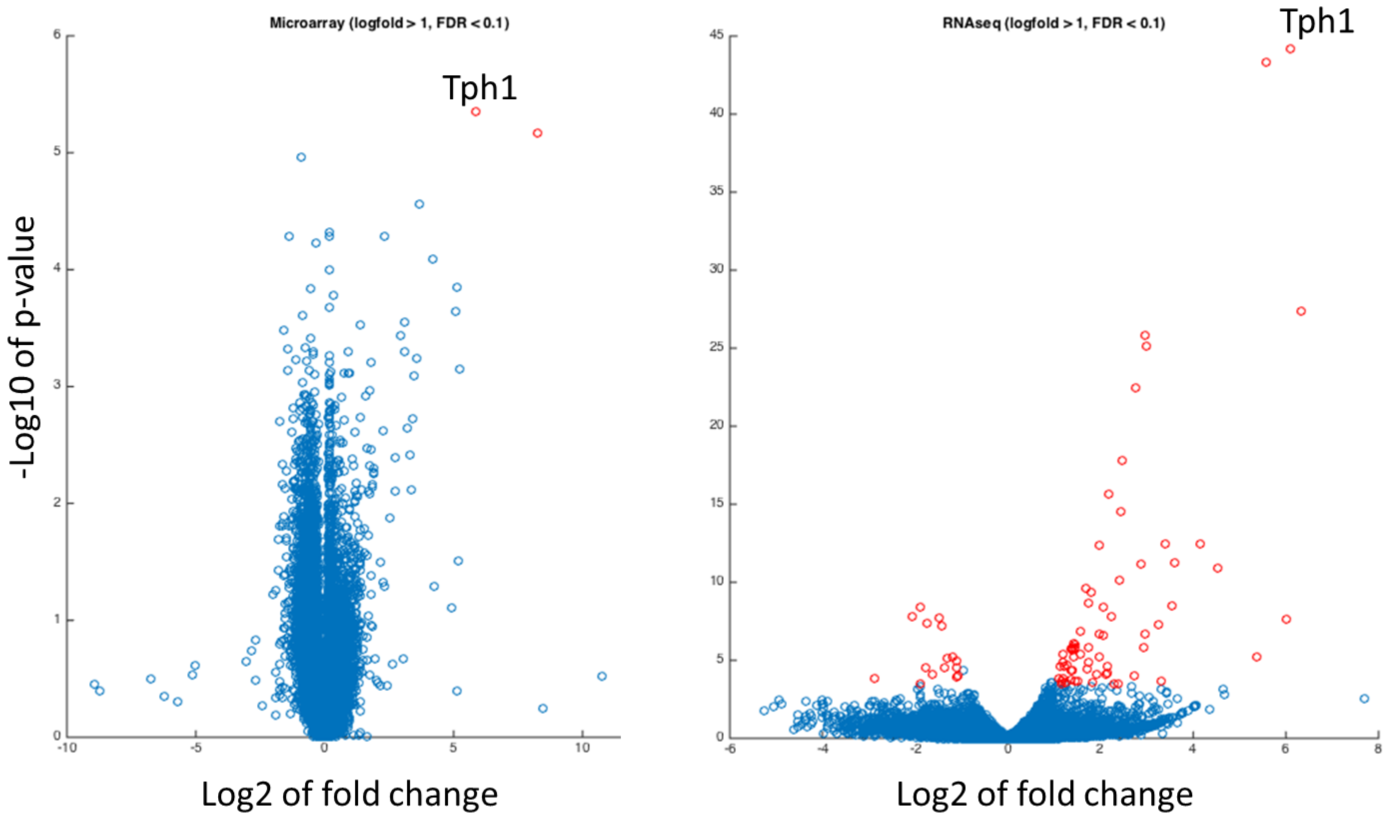

S1 Fig. Comparison of all coding and non-coding genes from NCode array and RNASeq. Both microarray and RNA-seq data showed that Tph1 was the most significantly differentially expressed gene. However, according to our stringent statistical analysis, RNA-seq displayed higher sensitivity in detecting differentially expressed lncRNAs compared to the NCode array.
