# Supplementary material for: Long noncoding RNAs are dynamically regulated during β-cell mass expansion in mouse pregnancy and control β-cell proliferation in vitro
Source: PLoS One. 2017 Aug 10;12(8):e0182371. doi: 10.1371/journal.pone.0182371 (PMC5552087; doi:10.1371/journal.pone.0182371)
Supplement: S2 Fig — (PDF) [file pone.0182371.s002.pdf]

S2 Fig

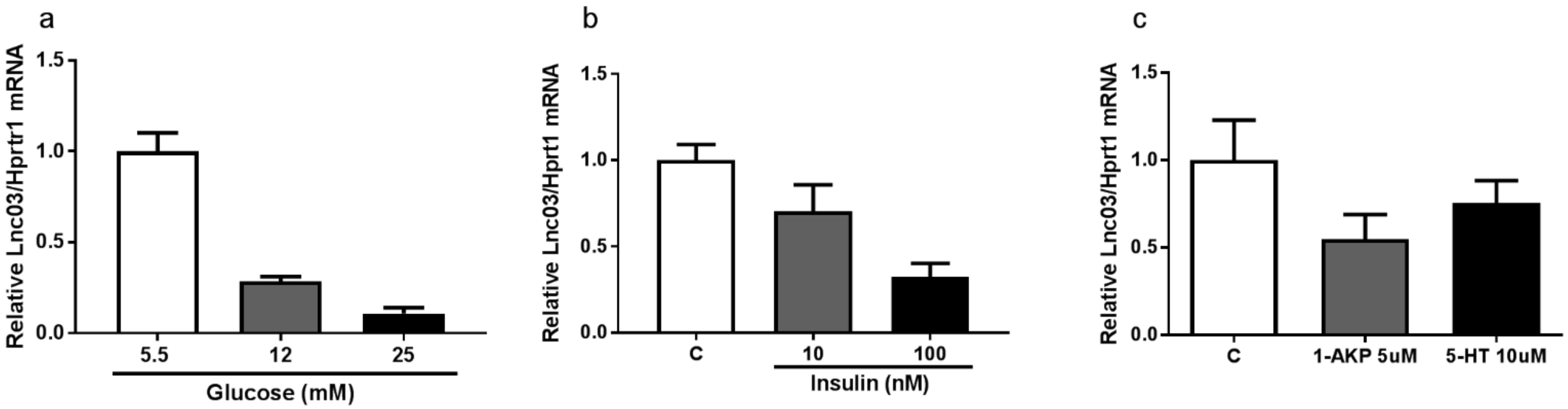

S2 Fig. Lnc03 in response to growth factors. Lnc03 expression in MIN6 cells in response to glucose (a), insulin (b) and the GSk3 inhibitor 1-AKP, as well as, 5-HT (c). Lnc03 expression was evaluated by RT-qPCR. Data are expressed as percentage of control (5.5 glucose or untreated cells (C).
