# Supplementary material for: Long noncoding RNAs are dynamically regulated during β-cell mass expansion in mouse pregnancy and control β-cell proliferation in vitro
Source: PLoS One. 2017 Aug 10;12(8):e0182371. doi: 10.1371/journal.pone.0182371 (PMC5552087; doi:10.1371/journal.pone.0182371)
Supplement: S3 Fig — (PDF) [file pone.0182371.s003.pdf]

S3 Fig

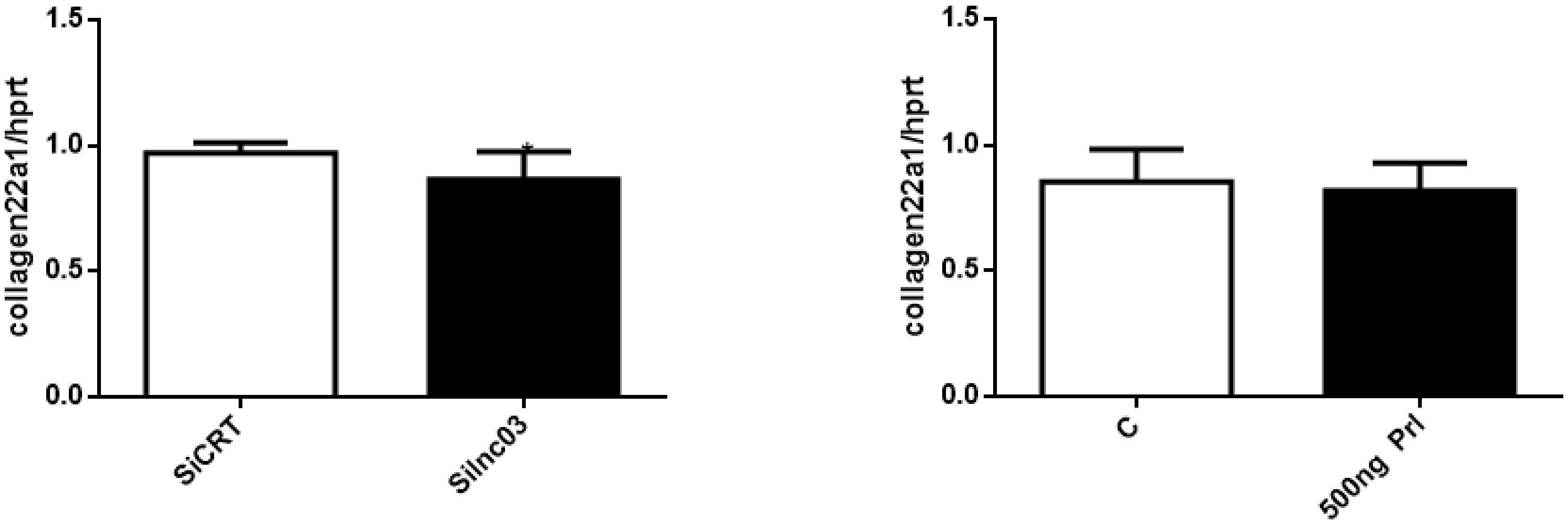

S3 Fig. Col22a1 expression in MIN6. Col22a1 expression in MIN6 cells (a) transfected with siRNA negative control (SiCRT) or SiLnc03 for 72h or treated with 500 ng/ml Prl for 24h. Col22a1 expression was evaluated by RT-qPCR. Data are expressed as house keeping gene (Hprt) ratios (mean±SEM), n=3. One-way ANOVA with Bonferroni post hoc test was applied to determine statistical significances (no differences).
