# Supplementary material for: Long noncoding RNAs are dynamically regulated during β-cell mass expansion in mouse pregnancy and control β-cell proliferation in vitro
Source: PLoS One. 2017 Aug 10;12(8):e0182371. doi: 10.1371/journal.pone.0182371 (PMC5552087; doi:10.1371/journal.pone.0182371)
Supplement: S4 Fig — (PDF) [file pone.0182371.s004.pdf]

S4 Fig

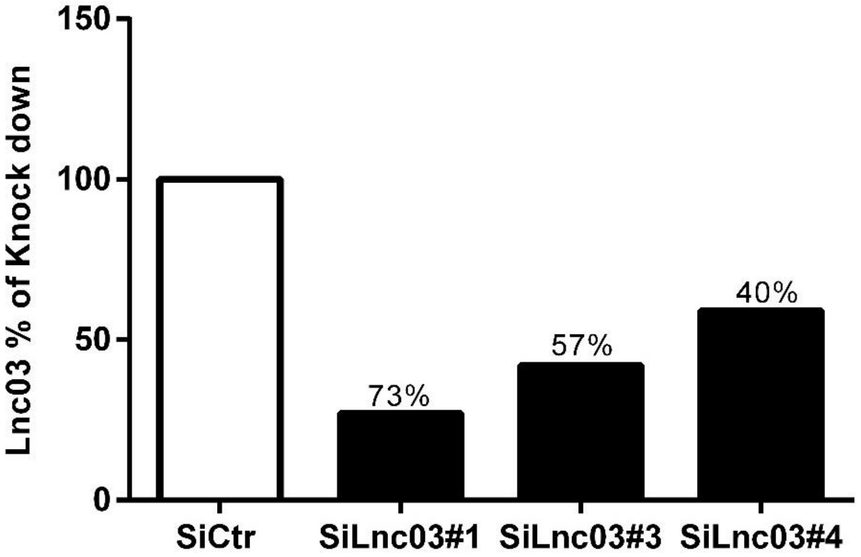

S4 Fig. Validation of Lnc03 knockdown in MIN6 cells. Lnc03 expression in MIN6 cells transfected with siRNA negative control (SiCtr, white bar) and three different SiRNA against Lnc03 (SiLnc03#1,#3 ,#4). Lnc03 expression was evaluated by RT-qPCR. Data are expressed as percentage of knockdown compared to SiCtr. b) Insulin secretion on MIN6 cells transfected with SiRNA negative control (SiCtr, white bar) and SiLnc03 (black bar).
