# Supplementary material for: Long noncoding RNAs are dynamically regulated during β-cell mass expansion in mouse pregnancy and control β-cell proliferation in vitro
Source: PLoS One. 2017 Aug 10;12(8):e0182371. doi: 10.1371/journal.pone.0182371 (PMC5552087; doi:10.1371/journal.pone.0182371)
Supplement: S5 Fig — (PDF) [file pone.0182371.s005.pdf]

S5 Fig. Analyses of cell cell cycle genes after Lnc03 knock down. MIN6 cells were transfected with siLnc03 or siCtr and subjected to 500ng/ml prolactin for 24h. Gene expression was normalized to siCtr treated cells. Differential gene expression was not enriched in any particular annotated group (Fisher's exact test).

S5 Fig

Gene Symbol	Protein class	Fold change	P-Value	Significant P-Value	Relative change
Cdkn3	G1 Phase & G1/S Transition	-1.35	0.026	✓	
Myb	G1 Phase & G1/S Transition	-1.47	0.060		
Gpr132	G1 Phase & G1/S Transition	-1.16	0.110		
Slfn1	G1 Phase & G1/S Transition	-1.14	0.110		
Cdk6	G1 Phase & G1/S Transition	-1.06	0.112		
Itgb1	G1 Phase & G1/S Transition	-1.18	0.126		
Chek1	G2 Phase & G2/M Transition	-1.18	0.151		
Birc5	G2 Phase & G2/M Transition	-1.14	0.180		
Ppm1d	G2 Phase & G2/M Transition	-1.08	0.549		
Wee1	M Phase	1.07	0.004	✓	
Cdc25c	M Phase	-1.25	0.005	✓	
Shc1	M Phase	-1.26	0.008	✓	
Cdc20	M Phase	-1.42	0.021	✓	
Ccna1	M Phase	-1.40	0.034	✓	
Terf1	M Phase	-1.18	0.075		
Cdk2	M Phase	-1.11	0.099		
Cdc25a	M Phase	-1.20	0.105		
Stmn1	M Phase	-1.05	0.111		
Brca2	M Phase	1.86	0.222		
Nek2	M Phase	-1.08	0.299		
Rad21	M Phase	-1.07	0.384		
Ran	M Phase	-1.05	0.489		
Ccnb1	M Phase	-1.03	0.651		
Stag1	M Phase	-1.02	0.821		
Smc1a	M Phase	1.01	0.847		
Cdk1	M Phase	1.00	0.922		
Aurkb	M Phase	1.00	0.934		
Gadd45a	Cell Cycle Checkpoint & Cell Cycle Arrest	-1.13	0.020	✓	
Casp3	Cell Cycle Checkpoint & Cell Cycle Arrest	-1.36	0.021	✓	
Atr	Cell Cycle Checkpoint & Cell Cycle Arrest	-1.40	0.021	✓	
Rb1	Cell Cycle Checkpoint & Cell Cycle Arrest	-1.40	0.028	✓	
Mad2l1	Cell Cycle Checkpoint & Cell Cycle Arrest	-1.26	0.067		
Cdkn2a	Cell Cycle Checkpoint & Cell Cycle Arrest	-1.26	0.079		
Dst	Cell Cycle Checkpoint & Cell Cycle Arrest	-1.27	0.095		
Notch2	Cell Cycle Checkpoint & Cell Cycle Arrest	-1.13	0.101		
Pmp22	Cell Cycle Checkpoint & Cell Cycle Arrest	-1.14	0.150		
Tsg101	Cell Cycle Checkpoint & Cell Cycle Arrest	-1.04	0.150		
Nbn	Cell Cycle Checkpoint & Cell Cycle Arrest	-1.05	0.162		
Hus1	Cell Cycle Checkpoint & Cell Cycle Arrest	-1.14	0.172		
Cdkn2b	Cell Cycle Checkpoint & Cell Cycle Arrest	-1.14	0.183		
Sfn	Cell Cycle Checkpoint & Cell Cycle Arrest	-1.06	0.188		
Rad9a	Cell Cycle Checkpoint & Cell Cycle Arrest	-1.18	0.247		
Mdm2	Cell Cycle Checkpoint & Cell Cycle Arrest	-1.08	0.294		
Pkd1	Cell Cycle Checkpoint & Cell Cycle Arrest	-1.10	0.349		
Cdkn1b	Cell Cycle Checkpoint & Cell Cycle Arrest	1.05	0.520		
Cks1b	Cell Cycle Checkpoint & Cell Cycle Arrest	1.04	0.600		
Ddit3	Cell Cycle Checkpoint & Cell Cycle Arrest	-1.04	0.639		
Chek2	Cell Cycle Checkpoint & Cell Cycle Arrest	1.05	0.771		
Cdk5rap1	Cell Cycle Checkpoint & Cell Cycle Arrest	1.01	0.864		
Cdkn1a	Cell Cycle Checkpoint & Cell Cycle Arrest	1.01	0.901		

Gene Symbol	Protein class	Fold change	P-Value	Significant P-Value	Relative change
Ccnd1	Regulation of the Cell Cycle	-1.30	0.008	✓	
Abl1	Regulation of the Cell Cycle	-1.29	0.024	✓	
Ccna2	Regulation of the Cell Cycle	-1.32	0.034	✓	
Aurka	Regulation of the Cell Cycle	-1.33	0.048	✓	
Bcl2	Regulation of the Cell Cycle	-1.28	0.066		
Ccnd3	Regulation of the Cell Cycle	-1.25	0.068		
Skp2	Regulation of the Cell Cycle	-1.12	0.081		
E2f2	Regulation of the Cell Cycle	1.12	0.082		
Ccnd2	Regulation of the Cell Cycle	-1.17	0.083		
E2f3	Regulation of the Cell Cycle	-1.16	0.132		
Ccnf	Regulation of the Cell Cycle	-1.11	0.135		
Ccne1	Regulation of the Cell Cycle	-1.21	0.138		
E2f1	Regulation of the Cell Cycle	1.06	0.299		
Tfdp1	Regulation of the Cell Cycle	-1.12	0.322		
Ccnb2	Regulation of the Cell Cycle	1.16	0.378		
E2f4	Regulation of the Cell Cycle	-1.17	0.422		
Cdk4	Regulation of the Cell Cycle	-1.54	0.864		
Ccnc	Regulation of the Cell Cycle	-1.01	0.925		
Atm	Negative Regulation of the Cell Cycle	-1.43	0.024	✓	
Trp53	Negative Regulation of the Cell Cycle	-1.23	0.231		
Brca1	Negative Regulation of the Cell Cycle	-1.10	0.341		
Rbl2	Negative Regulation of the Cell Cycle	-1.09	0.398		
Trp63	Negative Regulation of the Cell Cycle	-1.02	0.677		
Rbl1	Negative Regulation of the Cell Cycle	-1.02	0.821		
Mki67	Other	-1.37	0.015	✓	
Msh2	Other	-1.46	0.017	✓	
Cdc6	Other	-1.27	0.037	✓	
Cdc7	Other	-1.20	0.046	✓	
Rad17	Other	-1.18	0.051		
Mcm2	Other	-1.14	0.117		
Mre11a	Other	-1.48	0.290		
Mcm4	Other	-1.38	0.640		
Mcm3	Other	-1.03	0.711		
Rad51	Other	-1.02	0.792		
B2m	Housekeeping gene	-1.22	0.0527		
Gapdh	Housekeeping gene	-1.07	0.175		
Gusb	Housekeeping gene	-1.11	0.297		
Hsp90ab1	Housekeeping gene	-1.06	0.447		
Actb	Housekeeping gene	-1.05	0.487		
