# Supplementary material for: Long noncoding RNAs are dynamically regulated during β-cell mass expansion in mouse pregnancy and control β-cell proliferation in vitro
Source: PLoS One. 2017 Aug 10;12(8):e0182371. doi: 10.1371/journal.pone.0182371 (PMC5552087; doi:10.1371/journal.pone.0182371)
Supplement: S2 Table — (PDF) [file pone.0182371.s007.pdf]

S2 Table. **Taqman probes**

LncRNAs and reference gene	Taqman ID
Lnc01	Mm01343153_m1
Lnc02	Mm01234921_m1
Lnc03	Mm03976287_g1
Lnc04	Mm01249971_m1
Lnc05	Mm01264868_m1
Lnc06	AJT96JB
Hprt	Mm00446968_m1
