# Supplementary material for: Long noncoding RNAs are dynamically regulated during β-cell mass expansion in mouse pregnancy and control β-cell proliferation in vitro
Source: PLoS One. 2017 Aug 10;12(8):e0182371. doi: 10.1371/journal.pone.0182371 (PMC5552087; doi:10.1371/journal.pone.0182371)
Supplement: S3 Table — (PDF) [file pone.0182371.s008.pdf]

S3 Table. SiRNA against Lnc03 sense sequences

siRNAs	Sense Sequence
SiLnc03#1	CCAUCCAUGUAGUACAACAtt
SiLnc03#3	CCAUCUGACUUGUGAAGAAtt
SiLnc03#4	GUCCUUUAGUGGAUUCUCAtt
